# A Comparison of the Process of Remodeling of Hydroxyapatite/Poly-D/L-Lactide and Beta-Tricalcium Phosphate in a Loading Site

**DOI:** 10.1155/2015/730105

**Published:** 2015-10-04

**Authors:** Hiroyuki Akagi, Hiroki Ochi, Satoshi Soeta, Nobuo Kanno, Megumi Yoshihara, Kenshi Okazaki, Takuya Yogo, Yasuji Harada, Hajime Amasaki, Yasushi Hara

**Affiliations:** ^1^Division of Veterinary Surgery, Department of Veterinary Science, Faculty of Veterinary Medicine, Nippon Veterinary and Life Science University, 1-7-1 Musashino, Tokyo 180-8602, Japan; ^2^Division of Veterinary Microbiology, Department of Veterinary Science, Faculty of Veterinary Medicine, Nippon Veterinary and Life Science University, 1-7-1 Musashino, Tokyo 180-8602, Japan; ^3^Division of Veterinary Anatomy, Department of Veterinary Science, Faculty of Veterinary Medicine, Nippon Veterinary and Life Science University, 1-7-1 Musashino, Tokyo 180-8602, Japan; ^4^Medical Research Group, Development Department, Takiron Co., Ltd., 7-1-19, Minatojima Minamimachi, Chuo-ku, Kobe 650-0047, Japan

## Abstract

Currently, the most commonly used bioresorbable scaffold is made of beta-tricalcium phosphate (*β*-TCP); it is hoped that scaffolds made of a mixture of hydroxyapatite (HA) and poly-D/L-lactide (PDLLA) will be able to act as novel bioresorbable scaffolds. The aim of this study was to evaluate the utility of a HA/PDLLA scaffold compared to *β*-TCP, at a loading site. Dogs underwent surgery to replace a section of tibial bone with a bioresorbable scaffold. After the follow-up period, the scaffold was subjected to histological analysis. The HA/PDLLA scaffold showed similar bone formation and superior cell and tissue infiltration compared to the *β*-TCP scaffold, as seen after Villanueva Goldner staining. Moreover, silver staining and immunohistochemistry for Von Willebrand factor and cathepsin K demonstrated better cell infiltration in the HA/PDLLA scaffold. The fibrous tissue and cells that had infiltrated into the HA/PDLLA scaffold tested positive for collagen type I and RUNX2, respectively, indicating that the tissue and cells that had infiltrated into the HA/PDLLA scaffold had the potential to differentiate into bone. The HA/PDLLA scaffold is therefore likely to find clinical application as a new bioresorbable scaffold.

## 1. Introduction

In the field of veterinary orthopedics, bone transplantation has been generally applied for the large bone defect associated with the surgery for bone tumors, trauma, or infection. Bone grafts include autografts, allografts, demineralized bone matrix, and bioresorbable scaffolds. Autografts have been the gold standard of bone grafting, because of the superior osteoconductivity and osteoinductivity achieved, although retrieving autograft tissue causes pain at the surgical site and poses an infectious risk, and there are limits to the amount of material that can be retrieved. Thus, the development of an ideal bioresorbable scaffold has been an ongoing focus in orthopedic surgery.

From the 1980s, researches for development of bioresorbable scaffolds which have osteoconductive, osteoinductive, and bioresorptive properties have advanced [1]. Recently, an artificial bone, constructed from calcium phosphate and bioresorbable polymers, has been developed and applied in the regeneration of bone and cartilage [2–5].

One new form of porous artificial bone is mainly composed of hydroxyapatite (HA) and *β*-tricalcium phosphate (*β*-TCP). These porous scaffolds are highly biocompatible and show good osteoconductivity [6]. On the other hand, porous scaffolds take a long time to be absorbed, and some of them demonstrate low osteoinductive properties [7]. In general, HA has superior biocompatibility compared to *β*-TCP but shows delayed resorption* in vivo* [8].

Polyglycolic acid (PGA) and polylactic acid (PLA) are typical bioresorbable polymers. These bioresorbable polymers can be used in various shapes, like rods, pins, plates, and screws [9]. In clinical cases, osteosynthetic implants made of PLA and PGA have been used for the treatment of fractures at various sites, such as the femoral head, the condyle of the femur, the condyle of the humerus, and the carpal bone [10]. However, these bioresorbable polymers generally show low bioactivity, and the rate of resorption tends to be dependent on the capacity of the material [6, 11].

After 2000, new bioresorbable scaffolds, composed of a combination of calcium phosphate and bioresorbable polymers, have been developed, in order to utilize the advantages of both types of materials and to compensate for their drawbacks [12, 13]. The aim of this combination was to achieve appropriate structural strength and direct union with the host bone and to match the processes of regeneration of bone and scaffold resorption. We previously reported that HA/poly-L-lactide (PLLA) achieved complete remodeling into cortical bone, but that this was not the case with PLLA only [14]. Shikinami et al. and Sai and Fujii reported that HA/poly-L/D-lactide (PDLLA), containing 70 wt% unsintered-HA particles in 30 wt% PDLLA, demonstrated superior biocompatibility and good bioresorption in the medullary cavity of the rabbit [15, 16]. The HA/PDLLA scaffold demonstrated good remodeling at an unloaded site, but the remodeling process at a loaded site was not then investigated.

The aim of this study was to evaluate the usefulness of the HA/PDLLA scaffold in a loaded site, by analyzing the remodeling process in comparison to that achieved with a *β*-TCP scaffold.

## 2. Material and Methods

### 2.1. Bioresorbable Scaffold

**Figure 1:**
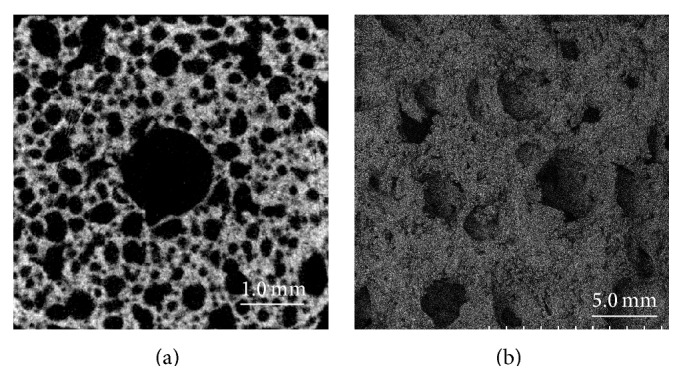
Images of micro-CT and scanning electron microscope (SEM). (a) The micro-CT image of HA/PDLLA scaffold. (b) The SEM image of HA/PDLLA scaffold.

The HA/PDLLA scaffold was composed of 70 wt% unsintered HA and 30 wt% PDLLA (Mv: 77 kDa; D/L: 50/50 mol%) matrix and was prepared by hot-compression moulding of nonwoven composite fibers. Manufacturing involved the scaffold fiber precipitation method, following the report by Shikinami et al. [15]. The HA/PDLLA scaffold (Comporus: Takiron, Osaka, Japan) had 70% porosity, a 40–480 *μ*m (average: 170 *μ*m) interconnected pore size, and 4.1 ± 0.4 MPa compressive strength [15]. The structure of the HA/PDLLA scaffold, identified using scanning electron microscope (SEM) and micro-CT, was shown in Figure 1. The control material, that is, the *β*-TCP scaffold (Osferion60; Olympus Terumo Biomaterials, Tokyo, Japan) had 60% porosity, 10 MPa compressive strength, and 100–165 *μ*m pore size. Both biomaterial scaffolds were prepared to a cuboid of size 10 × 10 × 15 mm, and the center area had a 3.0 mm hole.

### 2.2. Animals

Nine healthy male beagles were included in the study. All dogs were 11.4 ± 0.5 (mean ± SD) months of age at the beginning of the experiment and their average weight was 9.6 ± 0.9 kg. Surgical treatment and postoperative management of the dogs were performed in accordance with the Guidelines for Care and Use of Laboratory Animals of the Nippon Veterinary and Life Science University. Dogs were randomly divided into three groups: 1-, 3-, and 12-month group, with three dogs per group.

### 2.3. Surgical Treatment

**Figure 2:**
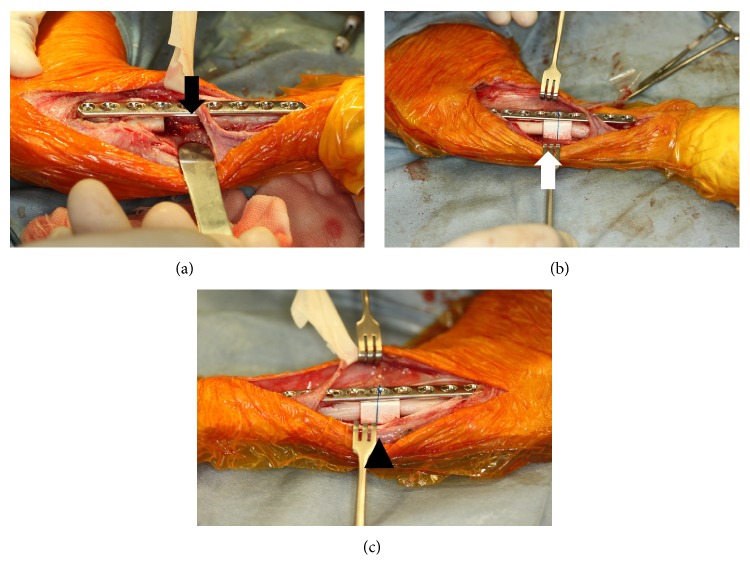
Images of surgery. (a) The central region of the tibia was removed using an oscillating bone saw (black arrow). (b) The HA/PDLLA composite was inserted into the space created (white arrow). (c) The *β*-TCP composite was inserted into the space created (black arrow head).

The dogs received preanesthetic injections of droperidol (0.25 mg/kg, i.m.), and general anesthesia was induced with propofol (7 mg/kg, i.v.). Each dog was intratracheally intubated, and anesthesia was maintained with isoflurane and oxygen. All anesthetized dogs received epidural injections of buprenorphine hydrochloride (5 *μ*g/kg) and bupivacaine hydrochloride (0.5 mg/kg). A surgical approach was made via the right medial side to expose the tibial diaphysis. The tibial length was measured prior to the operation; a 15-mm region of the central tibia was removed using an oscillating bone saw. A bridging plate fixation was applied using an 81 mm 9-hole locking compression plate (Synthes, SE) and *φ* 2.7 mm × 16 mm locking head screws (Synthes), using three screws to each of the proximal and distal region of the tibia (Figure 2(a)). Thereafter, the HA/PDLLA scaffold was inserted into the space (HP group; Figure 2(b)). The wound was closed routinely. The same procedure was used to implant a *β*-TCP scaffold into the left limb (TCP group; Figure 2(c)).

Animals were treated using a Robert–Jones bandage up to 14 days, with limited physical activity. Postoperative analgesic management was achieved by administration of buprenorphine (0.02 mg/kg, i.m.; q 12 h) for 14 days. Each dog received cefamezin (25 mg/kg, p.o., q 12 h) for 14 days after the operation. Animals were kept in a cage to rest during the follow-up period. Dogs were euthanized via administration of an overdose of sodium pentobarbital after the indicated time.

### 2.4. Radiographic Analysis

Craniocaudal radiographics were obtained at 1, 3, 6, 9, and 12 months. Radiographic analysis was conducted to assess the union of the bioresorbable scaffold and host bone, and the condition of the scaffold and the fixation system.

### 2.5. Ca_2_(PO_4_)_3_ Content

After euthanasia, all plates and screws were removed, and computed tomography (CT; Asteion, Toshiba Medical Systems Corporation, Tokyo, Japan) imaging performed. CT images of the full length of the tibia were obtained at 120 kV, 100 mA, and with 1.0 mm slice thickness, in a prone position. The DICOM data was retrieved to commercial image analysis software (Image J analysis software; NIH, Bethesda, MD). While obtaining CT images, standard bone mineral phantom (B-MAS 200, Kyoto Kagaku, Japan) was used to measure the CT value of both scaffolds. The Ca_2_(PO_4_)_3_ content was measured at the axial site of the central portion of the scaffold.

### 2.6. Histological Analysis

After CT analysis, both tibias of each dog were retrieved and fixed using 10% neutral buffered formalin for 7 days. A sample was cut at the axial center, and the proximal portion was decalcified with 10% EDTA. Thereafter, each specimen was embedded in paraffin. The central plane of the specimens was sectioned parallel to the sagittal plane at a thickness of 5 *μ*m, and stained hematoxylin and eosin (HE), and silver impregnation. The sections were randomly chosen and stained according to following protocol.

The distal portion was dehydrated in serial concentrations of ethanol (30, 50, 70, 80, 90, and 100% v/v; 2 days per concentration) and then embedded in LR White resin (London Resin Company Ltd., London, UK). The specimens were sectioned using a band saw (BS-300CP, EXAKT Apparatebau GmbH) parallel to the sagittal plane of the sample. Then, the surface of specimens was polished with diamond paper (MG-4000, EXAKT Apparatebau GmbH) and subjected to Villanueva Goldner (VG) staining, which was then observed under a light microscope.

### 2.7. Immunohistochemistry Staining

#### 2.7.1. Collagen Type 1 (COL1)

The proximal section of HA/PDLLA scaffolds and *β*-TCP scaffolds were cut into 5 *μ*m thick sections. The sections were stained with a polyclonal rat-anti-rabbit-COL1 (1 : 5000, Cosmo Bio, Tokyo, Japan) followed by a rabbit IgG antibody (CosmoBio), which served as secondary antibody. The complex was detected using 3,3′-diaminobenzidine, tetrahydrochloride (DAB; DAKO, Glostrup, Denmark).

#### 2.7.2. Von Willebrand (VW) Factor

The sections were stained with a polyclonal rat-anti-rabbit-VW factor (1 : 2000, Cosmo Bio) followed by anti-rabbit IgG antibody (Cosmo Bio), which served as secondary antibody. The complex was detected using DAB (DAKO).

#### 2.7.3. Cathepsin K

The sections were stained with a polyclonal human anti-goat cathepsin K antibody (1 : 300, Santa Cruz Biotechnology, Santa Cruz, CA) followed by anti-goat IgG antibody (Nichirei Biosciences, Inc., Tokyo Japan), as secondary antibody. The complex was detected using DAB (DAKO).

#### 2.7.4. RUNX2

The sections were stained with a polyclonal human anti-goat RUNX2 antibody (1 : 100, Santa Cruz Biotechnology), followed by anti-goat IgG antibody (Nichirei Biosciences, Inc.), as secondary antibody. The complex was detected using DAB (DAKO).

### 2.8. Qualitative and Quantitative Analysis

Qualitative and quantitative analysis was conducted as detailed in Table 1. Each image was taken using a BX51 microscope (Olympus, Tokyo, Japan) and saved as TIFF files. The images were analyzed using image J software (NIH, Bethesda, MD).

### 2.9. Statistical Analysis

Differences between HA/PDLLA and *β*-TCP were analyzed using the Mann-Whitney test. The temporal change in each scaffold was analyzed using Tukey's honestly significant difference test. These tests were performed using SPSS statistical software (SPSS, Japan Inc., Tokyo, Japan). Differences were considered significant at values of *p* < 0.05.

## 3. Results

### 3.1. Radiographic Analysis

**Figure 3:**
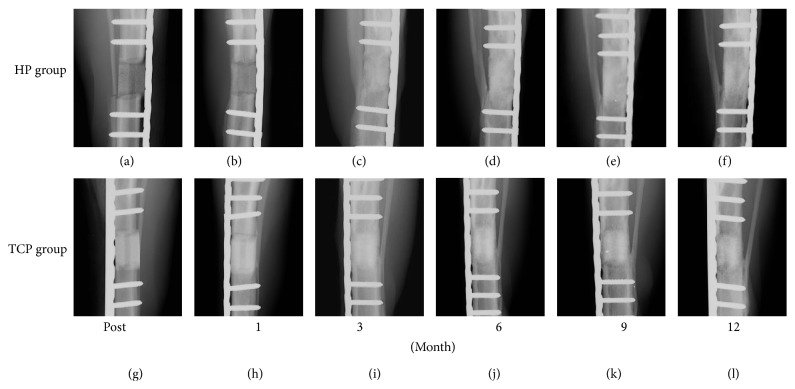
Craniocaudal view of the bioresorbable scaffold. ((a)–(f)) HP group. ((g)–(l)) TCP group. ((a), (g)) Postsurgery. ((b), (h)) 1 month. ((c), (i)) 3 months. ((d), (j)) 6 months, ((e), (k)) 9 months, and ((f), (l)) 12 months. Fibula fractures were found in six dogs, including three limbs in the TCP group and five limbs in the HP group, at 1- to 3-month follow-up. After 3 months, no fractures or refractures were observed. No fractures or displacement of the scaffold or fixation was seen.

Figure 3 shows the craniocaudal view of specimens, showing temporal radiographic changes. In the craniocaudal view, the TCP group indicated higher radiopacity compared to the HP group. At the 9-month follow-up, the border between the scaffold and host bone was unclear in the HP group (Figure 3(k)), indicating the continuousness of the HA/PDLLA scaffold and host bone; in contrast, the borders of the site and host bone could still be clearly seen at 12 months (Figure 3(l)).

### 3.2. Ca_3_(PO_4_)_2_ Content

**Figure 4:**
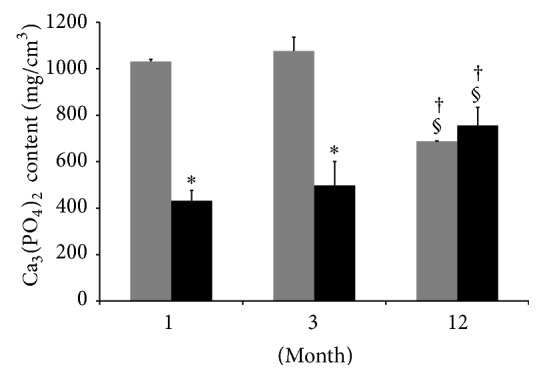
Ca_3_(PO_4_)_2_ content of the specimens. *β*-TCP (grey box), HA/PDLLA (black box). The 12-month HP group indicated a significantly higher Ca_3_(PO_4_)_2_ content compared to the 1- and 3-month groups. The 12-month TCP group had a significantly lower Ca_3_(PO_4_)_2_ content compared to the 1- and 3-month TCP groups; the TCP group also demonstrated a significantly higher Ca_3_(PO_4_)_2_ content compared to the HP group at 1 and 3 months. However, the TCP and HA groups were not significantly different at 12 months. *∗*: *p* < 0.05 versus TCP group. †: *p* < 0.05 versus 1 month. §: *p* < 0.05 versus 3 months.

The differences in Ca_3_(PO_4_)_2_ content are shown in Figure 4. The 12-month HP group indicated a significantly higher Ca_3_(PO_4_)_2_ content compared to the 1- and 3-month groups. The 12-month TCP group had a significantly lower Ca_3_(PO_4_)_2_ content compared to the 1- and 3-month TCP groups; the TCP groups also demonstrated a significantly higher Ca_3_(PO_4_)_2_ content compared to the HP group at 1 and 3 months. However, the TCP and HA groups were not significantly different at 12 months.

### 3.3. Histological Analysis

**Figure 5:**
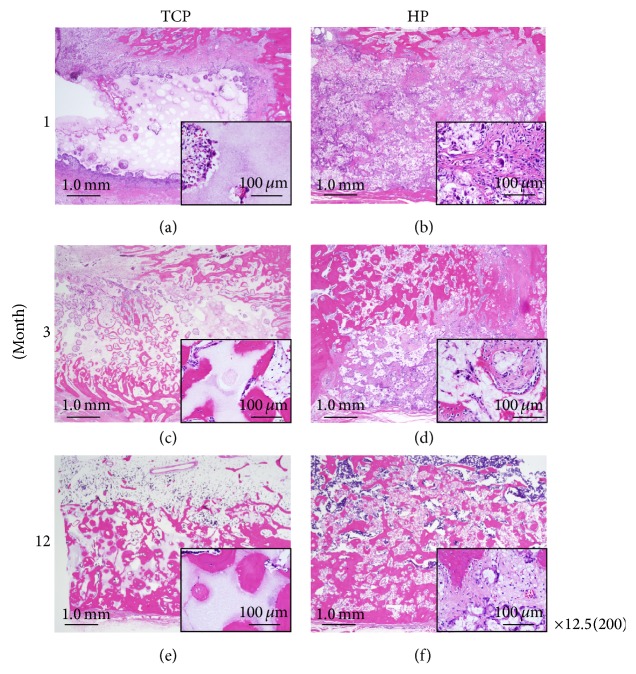
Tissue sections were stained with hematoxylin and eosin and viewed at ×12.5 and ×200 (insets) magnification. ((a), (c), and (e)) TCP group. ((b), (d), and (f)) HP group. ((a), (b)) 1 month. ((c), (d)) 3 months. ((e), (f)) 12 months. The HP group and TCP group showed the same level of bone formation. The HP group showed earlier scaffold resorption than did the TCP group. The process of remodeling was observed to be markedly different between the HP group and the TCP group. In particular, the HP group demonstrated significant fibrous tissue infiltration, whereas the TCP group did not. Scale bars: 1.0 mm and 100 *µ*m.

HE stained specimens are shown in Figure 5. At low magnification, the HP and TCP groups demonstrated the same level of bone formation, although the HP group showed earlier scaffold hydrolyzed than absorbed the TCP group. Moreover, the process of remodeling was observed to be markedly different between the HP group and the TCP group. Specifically, the HP group demonstrated significant fibrous tissue infiltration, whereas the TCP group did not.

### 3.4. Area of Bone Formation, Residual Scaffold, and Osteoid Tissue

**Figure 6:**
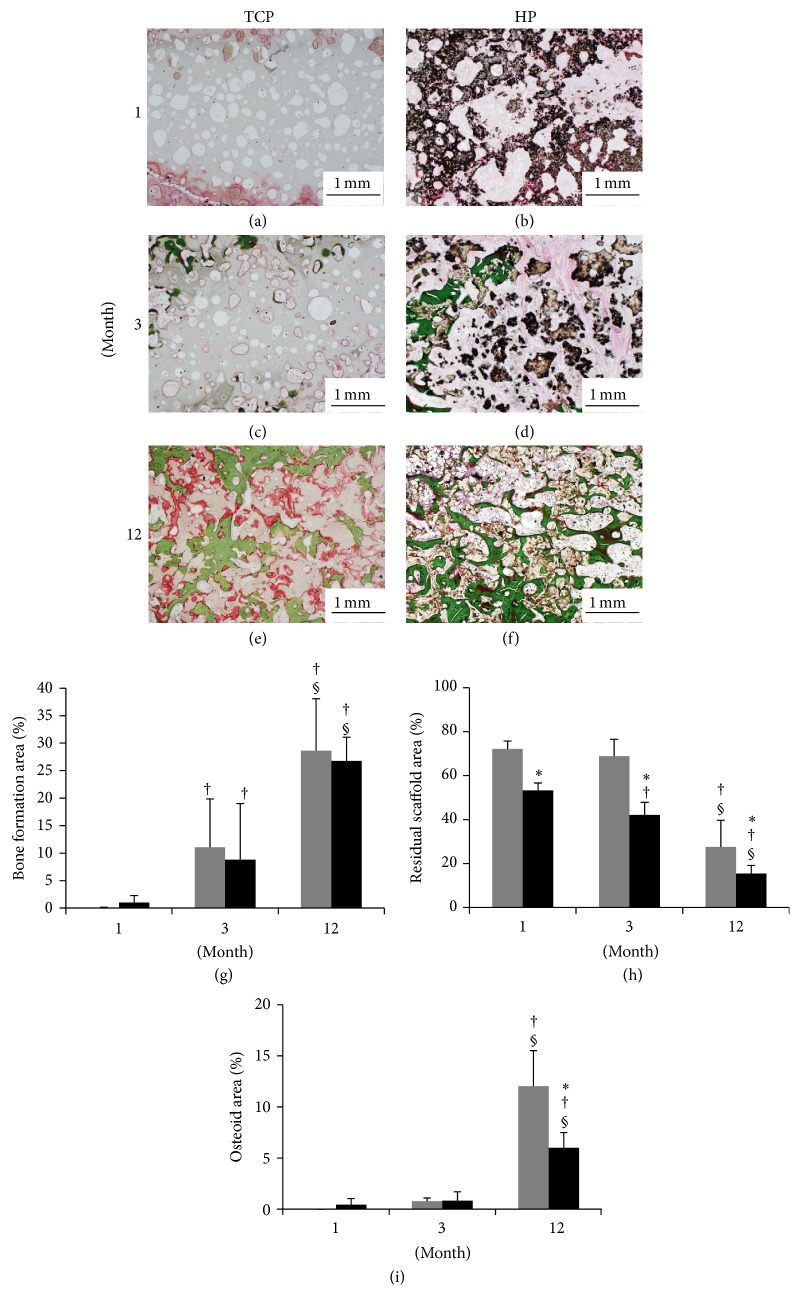
Tissue sections were stained with Villanueva Goldner (VG) stain and viewed at ×20 magnification. ((a), (c), and (e)) TCP group. ((b), (d), and (f)) HP group. ((a), (b)) 1 month. ((c), (d)) 3 months. ((e), (f)) 12 months. (g) Area of bone formation. (h) Area of residual composite. (i) Area of osteoid formation. *β*-TCP (grey box), HA/PDLLA (black box). (g) The HP and TCP groups showed the same level of bone formation, and both groups showed significant increases of bone formation over time. (h) The HP group represented superior scaffold resorption. (i) Both groups demonstrated a low level of osteoid formation at 1 and 3 months, although both groups showed significantly higher osteoid formation at 12 months. *∗*: *p* < 0.05 versus TCP group. †: *p* < 0.05 versus 1 month. §: *p* < 0.05 versus 3 months. Scale bars: 1.0 mm.

The areas representing bone formation (Figure 6(g)), residual scaffold (Figure 6(h)), and osteoid tissue (Figure 6(i)) was measured in specimens after VG staining. Both scaffolds indicated bone formation; in particular, the bone formation was significantly increased between 3 months and 12 months (Figure 6(g)). Both scaffolds indicated a lowering in the residual scaffold material over time; again, the residual scaffold was significantly reduced between 3 and 12 months (Figure 6(h)), with the HP group showing a significantly smaller area of residual scaffold than did the TCP group during the follow-up period. Both groups showed little osteoid tissue formation at 1 and 3 months, although both groups showed a significantly higher level of osteoid formation at 12 months (Figure 6(i)).

### 3.5. Fibrous Tissue

**Figure 7:**
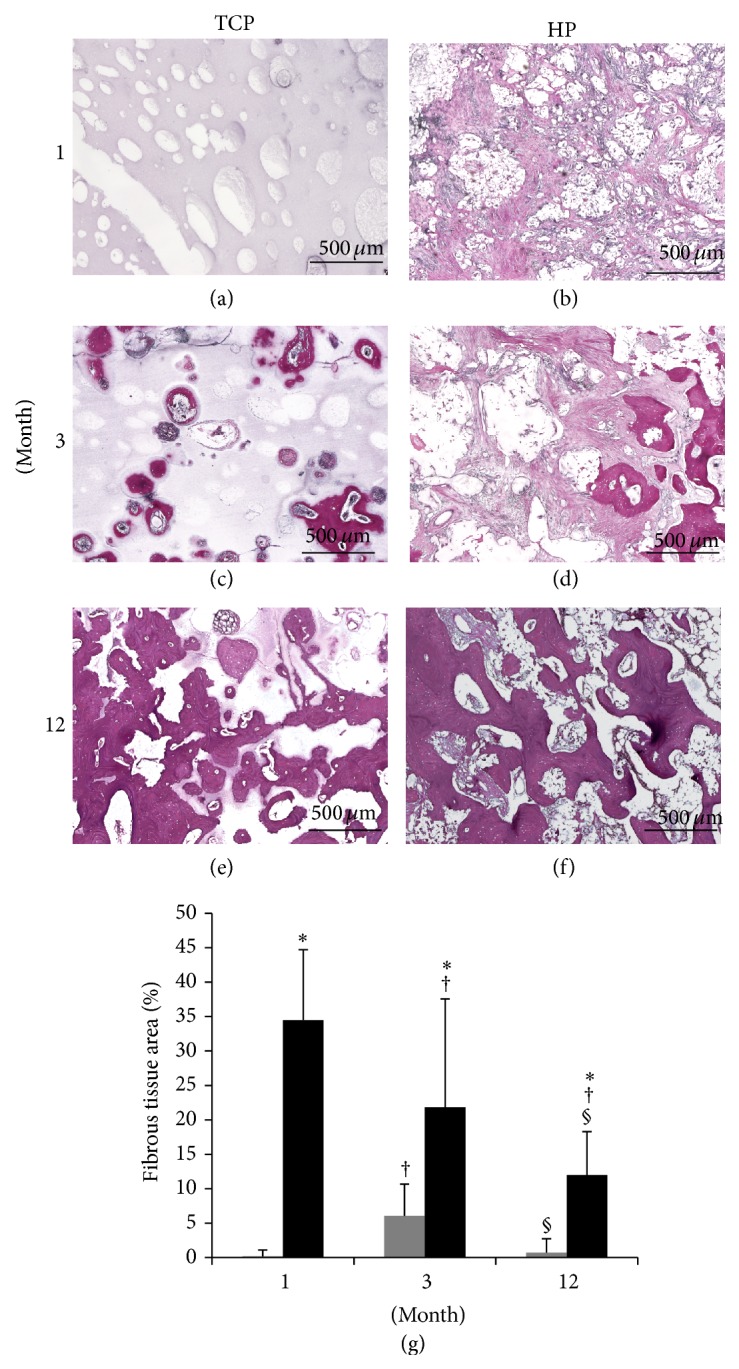
Tissue sections were stained by silver impregnation and observed at ×40 magnification. ((a), (c), and (e)) TCP group. ((b), (d), and (f)) HP group. ((a), (b)) 1 month. ((c), (d)) 3 months. ((e), (f)) 12 months. (g) Measurements of the infiltrated fibrous tissue. *β*-TCP (grey box), HA/PDLLA (black box). (g) The TCP group showed little fibrous tissue infiltration during the follow-up period. On the other hand, the HP group showed a stronger fibrous tissue infiltration than did the TCP group. *∗*: *p* < 0.05 versus TCP group. †: *p* < 0.05 versus 1 month. §: *p* < 0.05 versus 3 months. Scale bars: 500 *µ*m.

Specimens that had been stained by silver impregnation are shown in Figures 7(a)–7(f). The fibrous tissue areas are represented in Figure 7(g). There was little fibrous tissue infiltration in the TCP group (Figures 7(a), 7(c), and 7(e)), but the HP group showed greater infiltration of fibrous tissue (Figures 7(b), 7(d), and 7(f)). More specifically, there was less fibrous tissue infiltration in the caudal side compared to the cranial side (data not shown). There was a significant difference between the HA group and the TCP group during the follow-up period; the HP group showed a significant reduction in fibrous tissue infiltration over time.

### 3.6. COL1

**Figure 8:**
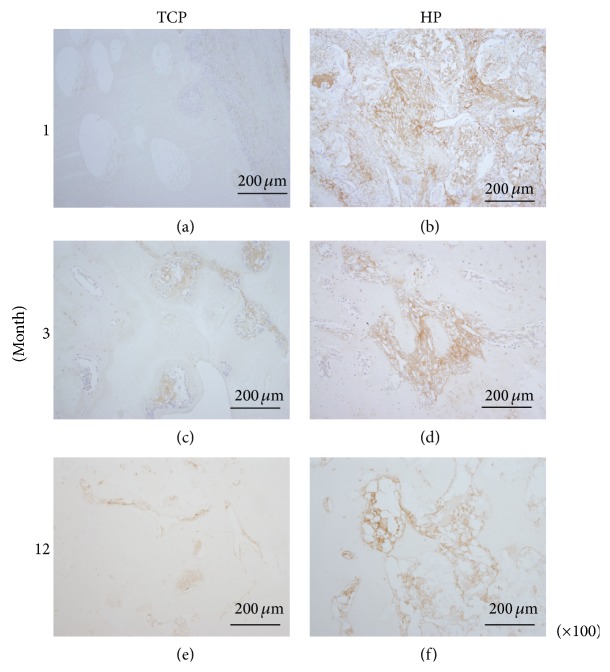
Tissue sections were stained for COL 1 and observed at ×200 magnification. ((a), (c), and (e)) TCP group. ((b), (d), and (f)) HP group. ((a), (b)) 1 month. ((c), (d)) 3 months. ((e), (f)) 12 months. Most of the infiltrated fibrous tissue in the bioresorbable scaffolds was positive for COL1. The HP groups showed COL1-positive tissue infiltration; particularly, the 1- and 3-month HP groups showed marked COL1-positive tissue infiltration. On the other hand, the TCP groups did not show much fibrous tissue infiltration. Scale bars: 200 *µ*m.

Specimens that had been stained for COL1 by immunohistochemistry are shown in Figures 8(a)–8(f). Most of the infiltrated tissue in the bioresorbable scaffolds was positive for COL1. As the TCP group did not show much fibrous tissue infiltration, there was little COL1-positive tissue in this group. In contrast, as the HP group demonstrated markedly more fibrous tissue infiltration, this group also showed more COL1-positive tissue. Specifically, the 1- and 3-month HP groups showed markedly more fibrous tissue infiltration, which was also positive for COL1 (Figures 8(b) and 8(d)).

### 3.7. Vessel Cavity Measurement

**Figure 9:**
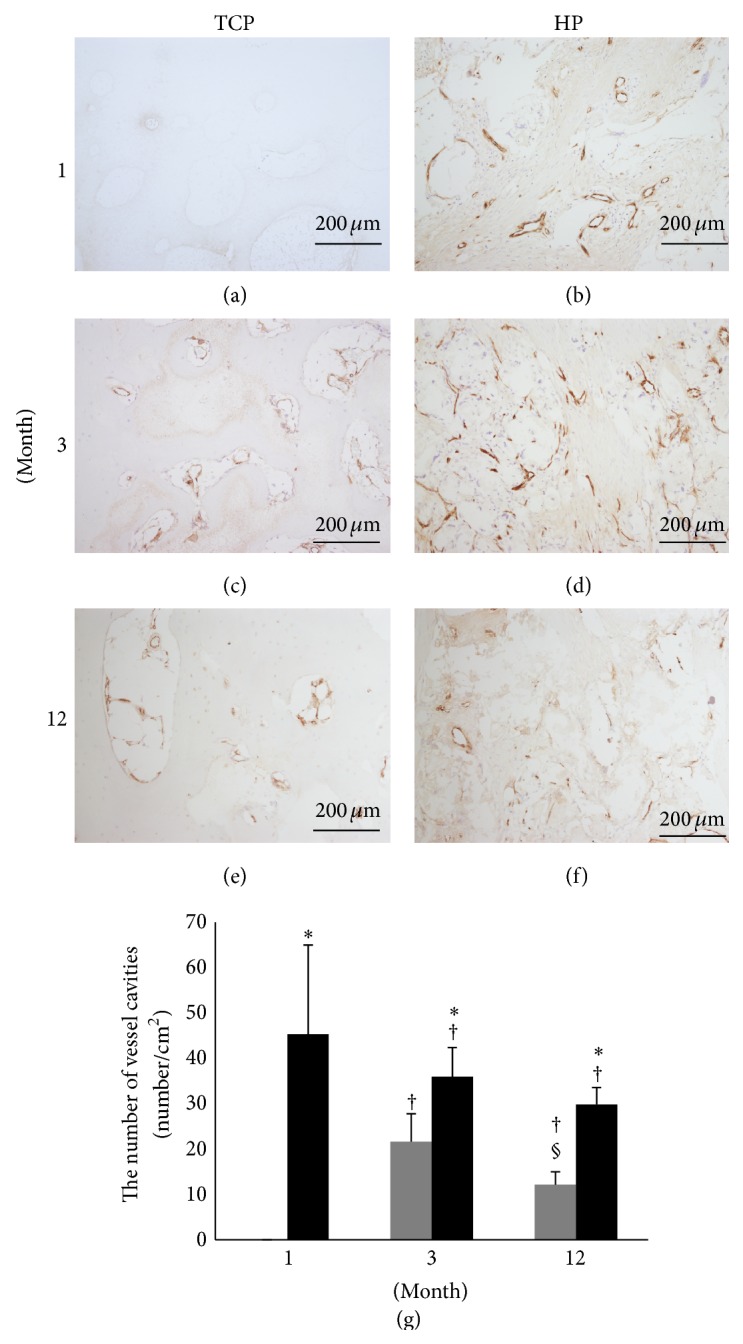
Tissue sections were stained for Von Willebrand (VW) factor and observed at ×100 magnification. ((a), (c), and (e)) TCP group. ((b), (d), and (f)) HP group. ((a), (b)) 1 month. ((c), (d)) 3 months. ((e), (f)) 12 months. (g) Measurement of the number of infiltrating vessels. *β*-TCP (grey box), HA/PDLLA (black box). Most of the vessel cavities were present in the infiltrated fibrous tissue; the HP groups showed significantly more vessel cavities than did the TCP groups during the follow-up period. *∗*: *p* < 0.05 versus TCP group. †: *p* < 0.05 versus 1 month. §: *p* < 0.05 versus 3 months. Scale bars: 200 *µ*m.

Figures 9(a)–9(f) show specimens stained for VW factor by immunohistochemistry. The number of vessel cavities positive for VW factor is shown in Figure 9(g). The HP groups showed a significantly higher number of vessel cavities than did the TCP groups during the follow-up period. Particularly, the 1-month HP group showed a markedly higher number of vessel cavities than did the 3- and 12-month HP groups. The TCP groups showed a significantly higher number of vessel cavities at 3 months compared to that at 1 month; however, this number decreased significantly by 12 months.

### 3.8. Osteoclast-Like Cells

**Figure 10:**
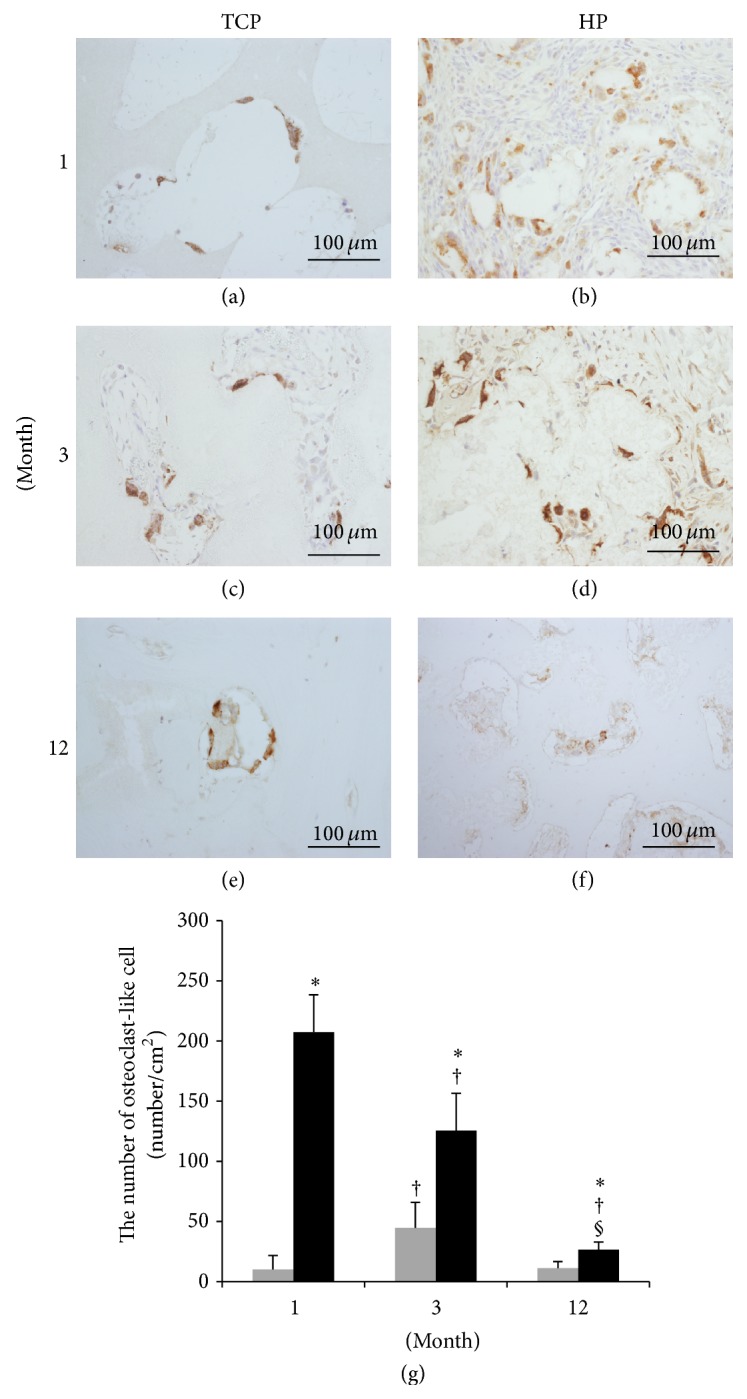
Tissue sections were stained for cathepsin K and viewed at ×200 magnification. ((a), (c), and (e)) TCP group. ((b), (d), and (f)) HP group. ((a), (b)) 1 month. ((c), (d)) 3 months. ((e), (f)) 12 months. (g) Number of osteoclast-like cells present after implantation of bioresorbable materials. *β*-TCP (grey box), HA/PDLLA (black box). The number of cathepsin K-positive cells was measured. We defined osteoclast-like cells as cells that were cathepsin K-positive and possessed more than five nuclei. HP groups showed significantly more osteoclast-like cells compared to the TCP groups during the follow-up period. In the HP group, the positive cells reduced significantly over time. In the TCP group, the 3-month group showed a significant more cathepsin K-positive cells compared to the 1- and 12-month groups. *∗*: *p* < 0.05 versus TCP group. †: *p* < 0.05 versus 1 month. §: *p* < 0.05 versus 3 months. Scale bars: 100 *µ*m.

Cathepsin K-positive specimens are shown in Figures 10(a)–10(f), and the results of measurement of the positive regions are shown in Figure 10(g). The HP group showed significantly more cathepsin K-positive cells than the TCP group during the follow-up period. In the HP group, the positive cells reduced significantly over time. In the TCP group, the 3-month group showed significantly more cathepsin K-positive cells compared to the 1- and 12-month groups.

### 3.9. RUNX2

**Figure 11:**
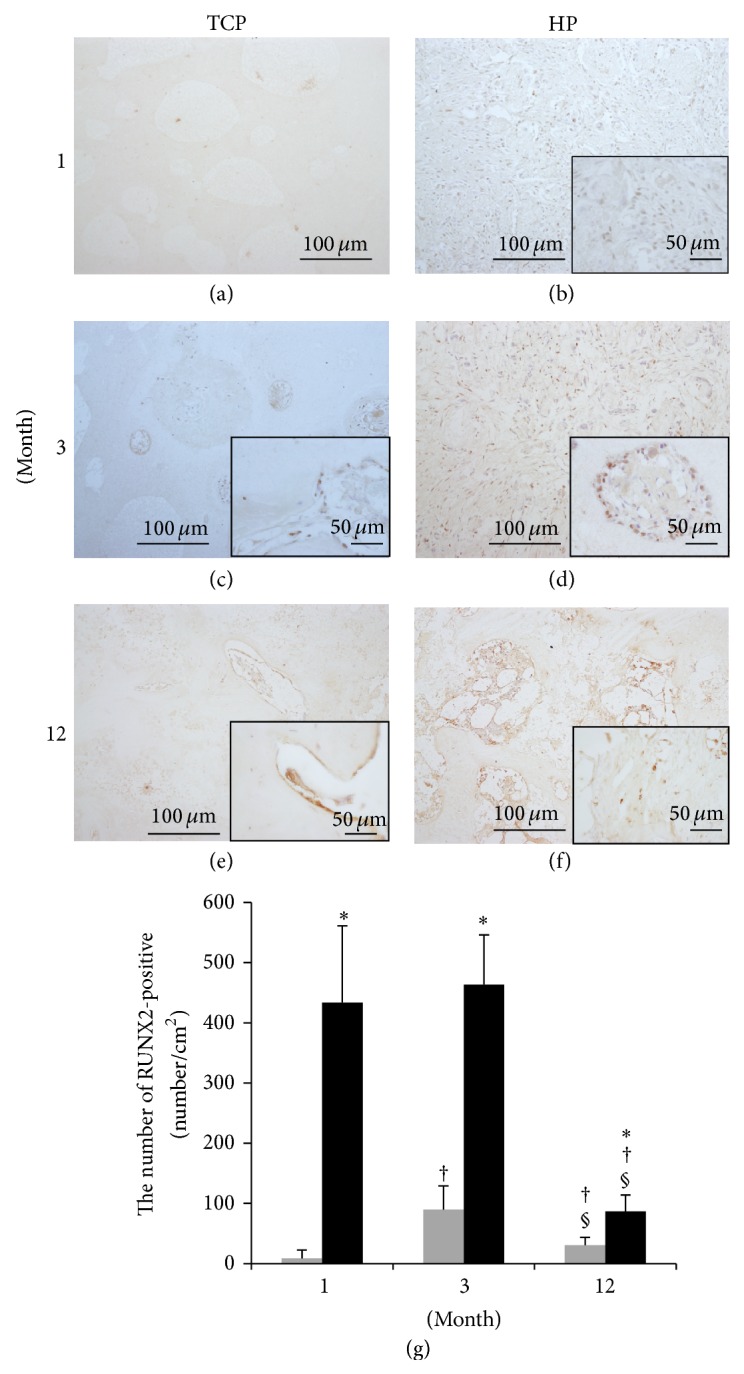
Tissue sections were stained for RUNX2 and viewed at ×100 and ×400 (insets) magnification. ((a), (c), and (e)) TCP group. ((b), (d), and (f)) HP group. ((a), (b)) 1 month. ((c), (d)) 3 months. ((e), (f)) 12 months. (g) The number of cells positive for RUNX2. *β*-TCP (grey box), HA/PDLLA (black box). Most of the spindle-shaped cells that were frequently observed in the HP group were positive for RUNX2. The HP group showed significantly more RUNX2-positive cells than did the TCP group during the follow-up period. In the HP group, specimens at 1 and 3 months demonstrated significantly more RUNX2-positive cells compared to the 12-month group. In the TCP group, the 3-month group demonstrated significantly more RUNX2-positive cells compared to the 1- and 12-month groups. *∗*: *p* < 0.05 versus TCP group. †: *p* < 0.05 versus 1 month. §: *p* < 0.05 versus 3 months. Scale bars: 100 *µ*m and 50 *µ*m.

The RUNX2-positive specimens are shown in Figures 11(a)–11(f) and the results of measurement of the corresponding areas are shown in Figure 11(g). Most of the spindle-shaped cells that were often observed in the HP group were positive for RUNX2. The HP group showed significantly more RUNX2-positive cells compared to the TCP group during the follow-up period. In the HP group, specimens at 1 and 3 months demonstrated significantly more RUNX2-positive cells compared to the 12-month group. In the TCP group, the 3-month group showed significantly more RUNX2-positive cells than did the 1- and 12-month groups.

## 4. Discussion

The aim of this study was to evaluate the usefulness of the HA/PDLLA scaffold compared to *β*-TCP scaffold, which is currently considered the most popular bioresorbable scaffold. The *β*-TCP scaffold has been reported to have superior osteoinduction and osteoconduction [16, 17]. Cutright et al. previously reported that implantation of *β*-TCP scaffold into rat tibia resulted in 95% of the scaffold being absorbed and formation of a medullary cavity after 48 days [18]. These results indicated that the *β*-TCP scaffold demonstrates good bioresorptive qualities, although the TCP group retained a clear scaffold form during the follow-up period. This is influenced by the scaffold porosity and pore size [19]. On the other hand, in our study, the border between the HA/PDLLA scaffold and host bone was not recognizable by 9 months. Examination of *β*-TCP scaffold implanted into rabbit femur resulted in a gradual increase in radiolucency [20]. In our study, the radiolucency gradually decreased, until the radiolucency of the *β*-TCP scaffold and the host bone was similar at 9 months. Differences in the species and examination procedures used may be responsible for differences in each result. However, obtaining equal radiolucency with the host cortical bone was a common result between previous reports and this study.

Upon histological analysis, the HP group and the TCP group indicated equal bone formation. Although the feature of bone formation were different between HA/PDLLA scaffold and *β*-TCP scaffold, the HP group showed significantly less residual scaffold than did the TCP group. Moreover, the HA/PDLLA scaffold indicated superior utility compared to the TCP scaffold. The HP group showed strong fibrous tissue infiltration, while the TCP group showed little fibrous tissue infiltration. These results indicated a difference in the remodeling process between the HA/PDLLA scaffold and *β*-TCP scaffold. In a study in which *β*-TCP scaffold was implanted into the canine dorsal region, the pattern of bone formation suggested intramembrane ossification [21]. In this study, the results obtained using the *β*-TCP scaffolds indicated slight fibrous tissue infiltration and significant osteoid tissue formation at 12 months. Thus, the remodeling process observed in this study was likely to involve intramembrane ossification. On the other hand, the HP group showed marked fibrous tissue infiltration, which gradually became calcified.

The VW factor is specifically expressed in vascular endothelial cells [22, 23]. Vessel formation plays an important role in both endochondral ossification and intramembrane ossification and for delivery of some cytokines, oxygen, nutrition, and various cells from the host site to the site of the bioresorbable scaffold [24, 25]. Moreover, lack of a blood supply causes delayed healing and lack of union at the fracture site [26]. In our study, the HP group demonstrated a significantly higher degree of vessel formation compared to that in the TCP group.

The results of silver staining and VW factor immunohistochemistry indicated that the HA/PDLLA scaffold demonstrated superior infiltration compared to the *β*-TCP scaffold.

The vessel cavity formed in the scaffold transports various types of cells. In particular, infiltration of osteoclast-like cells, which plays a role in scaffold resorption, is very important. Chazono et al. previously reported that bone formation occurs after scaffold resorption [27]. Cathepsin K is expressed in osteoclast cells [28–30]. The HP group showed a significantly higher number of cathepsin K-positive cells compared to the TCP group; this indicated that the HA/PDLLA scaffold was more likely to be resorbed by osteoclast-like cells than was the *β*-TCP scaffold. Additionally, more PDLLA is degraded by hydrolysis, so that the HA/PDLLA scaffold is likely to be useful in patients with osteopetrosis and diabetes [31].

The infiltrated fibrous tissue was positive for COL1 and most of the infiltrated cells were positive for RUNX2. RUNX2 plays a role in differentiation of mesenchymal cells into osteoblasts, activation of COL1 expression, and is also related to vessel formation [32–36]. RUNX2 influences not only bone formation, but also bone resorption [37–39]. Compared to the TCP group, the HP group had significantly higher numbers of RUNX2-positive cells throughout the entire follow-up period, suggesting that both bone formation and the remodeling process of bone resorption had been activated. The size of pore and interconnection pore were critical factors for biomaterial scaffolds [40–43]. Tsuruga et al. reported that for cell adhesion, differentiation, growth of osteoblasts, and vascularization, the optimal pore size was approximately 300–400 *μ*m [40]. Lu et al. recommended the favorable interconnection pore size to be over 50 *μ*m, while Xiao et al. reported that the 150 *μ*m interconnection pore size showed significant greater vascularization compared to 100 and 120 *μ*m [43, 44]. From these researches, it can be said that the HA/PDLLA scaffold was sufficient in both the pore size and interconnection pore size.

In generally, a bioresorbable scaffold does not have sufficient mechanical strength to be used at a loading site. However, Liu et al. reported that it was possible to apply bioresorbable scaffolds to a loading site while using a proper fixation system, although they reported that using *β*-TCP-only scaffold did not result in good bone formation [45]. In other words, a wide range of bone injuries could not be healed by transplantation of only cancellous bone [45]. In our study, using a particular bioresorbable scaffold alone resulted in good bone formation. We hypothesized that this was due to the animal species used, the porosity and pore size of the material used, and the medullary-like cavity created in the bioresorbable scaffold. In a previous study of a bioresorbable scaffold implanted subcutaneously into rabbit, cartilage tissue and chondrocytes were observed by 3 months [46]; however, we did not observe these features in our study, and the reason for this is not immediately clear.

Ozawa et al. reported a study in which 167 clinical cases received *β*-TCP scaffold implants and observed good remodeling radiologically [47]. However, the *β*-TCP scaffold could not be remolded easily to replace the defect site. In contrast, the HA/PDLLA scaffold could be formed into various shapes, using a scalpel and thermal modification, to fill the defect site optimally [15, 46], thereby offering easy intraoperative manipulation by the surgeon.

## 5. Conclusion

The results of this study showed that the HA/PDLLA scaffold is equal to the *β*-TCP scaffold in terms of bone formation and shows fine hydrolysis and operability. This study used 12 months as the follow-up period, but neither scaffold had achieved complete replacement by that period. The residual scaffold evoked pain and dysfunction. The result of immunohistochemistry staining showed that the HA/PDLLA scaffold had significantly higher infiltration and activation of the bone remodeling process than did the *β*-TCP scaffold; thus, the HA/PDLLA scaffold indicated a lower risk of residual scaffold. It is likely that the HA/PDLLA scaffold will find clinical application as a new bioresorbable scaffold.

## Figures and Tables

**Table 1:** Qualitative and quantitative analysis method.

Staining method	Magnification	Number of views	Aim
Silver impregnation	40	6	Area of fibrous tissue
COL 1	100	1	Identifying the fibrous tissue
Von Willebrand	100	10	Number of vessel cavities
Cathepsin K	200	10	Number of osteoclast-like cells
Runx2	200	10	Number of positive cells
VG staining	20	2	Bone, residual composite, osteoid
